# The Role of Dentate Gyrus Neurogenesis in Neuropsychiatric Disorders

**DOI:** 10.1155/2013/584382

**Published:** 2013-06-20

**Authors:** M. Julia García-Fuster, Justin S. Rhodes, Chitra D. Mandyam

**Affiliations:** ^1^University Research Institute on Health Sciences (IUNICS), University of the Balearic Islands, 07122 Palma de Mallorca, Spain; ^2^Department of Psychology, University of Illinois, Champaign, IL 61820, USA; ^3^Committee on the Neurobiology of Addictive Disorders, The Scripps Research Institute, La Jolla, CA 92037, USA

The special issue reviews the most recent developments in dentate gyrus (DG) neurogenesis with regard to neuropsychiatric disorders. A few laboratories worldwide have contributed to this special issue, and their contributions have showcased the current efforts in understanding the functional significance of DG neurogenesis in depression, psychosis, and addiction. Additionally, a promising therapeutic strategy for treating radiation therapy disorders is reviewed. The review and research articles in this special issue indicate that DG neurogenesis can be conceptualized as a form of plasticity and that the alterations in DG neurogenesis can predict a spectrum of disorders associated with the hippocampus. Highlights from the papers in the special issue are summarized below.

*Introduction*. The hippocampus is tactically situated in the brain such that it has reciprocal connections to several other regions involved in emotional behaviors, such as stress responses and positive and negative reinforcement. Alterations in these emotional behaviors can be considered precursors for the development of neuropsychiatric disorders, such as depression, schizophrenia, and drug addiction. Adult neurogenesis in the DG of the hippocampus is a new undisputed form of spontaneous plasticity, and most of the emotional behaviors implicated in neuropsychiatric disorders are known to inhibit DG neurogenesis. Therefore, the normalization of impaired DG neurogenesis during recovery or abstinence may be required to initiate normal neuroplasticity in the hippocampus, which could be critical for recovery. Contributions to this special issue include six review papers and one research paper that focus on the role of DG hippocampal neurogenesis in the etiology and treatment of three major neuropsychiatric disorders—depression, psychosis, and addiction—in human or animal models. Each paper was reviewed by at least two reviewers and revised according to the reviewers' comments.

*Summary*. Major depression is among the most prevalent psychiatric disorders, constituting the first leading cause of years lived with disability. In addition to the well-known monoamine neurotransmitter hypothesis of depression, many parallel processes exist that play a role in depression, such as alterations in neurotrophic factors (e.g., brain-derived neurotrophic factor (BDNF)) and the regulation of adult hippocampal neurogenesis. Three papers in this special issue discuss the neurogenesis hypothesis of depression, which postulates that the generation of new neurons in the adult DG is involved in the etiology and treatment of depression. The paper by H. Jun et al. reviews the literature on the effects of animal models of depression on adult hippocampal neurogenesis. They conclude that decreased neurogenesis is not associated with risk in the development of depressive behavior and speculate that newborn cells may be a major contributor to normalizing or ameliorating the disease state rather than being causally involved in the etiology of depression. The paper reviews possible therapeutic interventions to ameliorate depression symptoms that involve an increase in DG neurogenesis regulation. Nonclassic antidepressant methods (e.g., electroconvulsive therapy and deep brain stimulation) and chronic treatment with classic antidepressants and exercise are shown to stimulate neurogenesis in the DG. The molecular targets that participate in or mediate the antidepressant-induced increase in DG neurogenesis are also reviewed in this paper, such as the involvement of BDNF and vascular endothelial growth factor (VEGF), neurotrophic factors that regulate the *microniche* of adult neurogenesis. The paper by F. Pilar-Cuéllar et al. reviews the neurotrophic/plasticity hypothesis of depression by discussing basic and clinical studies that ascertained the role of intracellular signaling cascades in the regulation of neural proliferation and plasticity. They focus their review on hippocampal modulation by different types and subtypes of serotonergic receptors and trophic factors (e.g., BDNF and VEGF) through intracellular signaling pathways (e.g., cAMP, Wnt/*β*-catenin, and mTOR). The paper by S. R. Wainwright and L. A. M. Galea reviews the neural plasticity theory of depression by focusing on the potential role of DG neurogenesis and a cell adhesion molecule, the polysialylated form of neural cell adhesion molecule (PSA-NCAM). PSA-NCAM appears to be an interesting target for future studies because it plays a fundamental role in mediating the broad effects of antidepressant treatment across multiple forms of neural plasticity and appears to function at the confluence of prevailing theories of depression.

Schizophrenia is a complex psychiatric disorder with an onset during early adulthood or adolescence. To clarify the mechanisms that underlie the onset of the illness, clinicians are studying the early phases of psychosis that might transition to schizophrenia spectrum disorder. The literature on neurogenesis-relevant research on postmortem human tissues from schizophrenia patients and animal models shows diminished cell proliferation or altered molecular markers involved in the regulation of neurogenesis. Typical and atypical antipsychotics have been described to differentially regulate cell proliferation/neurogenesis, reflected by their actions, mechanisms, primary effects, and side effects. The paper by G. Keilhoff et al. reviews the effects of typical and atypical antipsychotic drugs on neurogenesis in animal models that reflect neurodevelopmental aspects of schizophrenia. A different approach to the study of the role of DG neurogenesis in schizophrenia is described in the paper by H. Hagihara et al., which reviews the literature that describes a phenomenon defined as an “immature dentate gyrus” (iDG). This phenomenon is characterized by arresting almost all neurons in the DG at a pseudoimmature state at both the molecular and electrophysiological levels and was identified in several strains of genetically engineered mutant mice with abnormal behaviors that are characteristic of neuropsychiatric disorders. It was first discovered in heterozygous knockout mice with a null mutation in a synaptic plasticity molecule (CaMKII) relevant to schizophrenia. The iDG mouse model has been associated with abnormal behaviors related to schizophrenia and other neuropsychiatric disorders, suggesting the importance of the adequate maturation and integration of adult-generated neurons into the hippocampal circuit for normal cognitive and emotional development. Interestingly, iDG-like phenomena have been observed in postmortem brains in patients with schizophrenia and bipolar disorder, and it has been proposed as a potential brain endophenotype that may be useful for classifying these disorders.

Drug addiction is a chronic relapsing disorder characterized by compulsive drug-seeking and -taking behavior and a loss of the ability to control drug intake. Hippocampal plasticity likely plays an important role in maintaining addictive behavior and/or relapse. The role of DG neurogenesis in drug addiction is discussed in two papers in this special issue. The paper by H. Jun et al. reviews selected prototype animal studies that investigated the effects of drug addiction (i.e., cocaine and alcohol) on adult hippocampal neurogenesis. Abused drugs are potent negative regulators of adult DG neurogenesis and as a result may impair cognitive function. The research article by M. P. Hicks et al. investigates the effects of extinction of heroin-seeking behavior on DG immature neurons. The authors show that extinction training following heroin self-administration increases the number of doublecortin-positive immature neurons selectively in the dorsal regions of the DG. They also present evidence that inhibition of cell proliferation in the DG increases responding during extinction, which is indicative of impaired extinction learning. These findings support the hypothesis that immature neurons in the DG may play a functional role in certain learning and memory processes, including the extinction of drug-seeking behavior. Diminished neurogenesis in the DG by drugs of abuse may play a causal role in certain cognitive deficits frequently observed in drug addicts

Hippocampal structure and function have been closely studied with regard to cognitive and mental function. The paper by S. P. Rodgers et al. reviews the role of exercise in ameliorating the suppression of cell proliferation and cognitive impairments observed in adulthood following pediatric radiotherapy. Ionizing radiation has been shown to disrupt brain development and suppress cell proliferation by killing the brain's actively dividing neural stem cells. This disrupted brain plasticity is believed to contribute to cognitive impairments. Therefore, the authors discuss the benefit of exercise as one potential treatment that is able to enhance both cell proliferation and neural plasticity needed for optimal cognitive performance.

